# DNA methylation status of *TBX20* in patients with tetralogy of Fallot

**DOI:** 10.1186/s12920-019-0534-3

**Published:** 2019-05-28

**Authors:** Juan Gong, Wei Sheng, Duan Ma, Guoying Huang, Fang Liu

**Affiliations:** 1 Children Hospital of Fudan University, Shanghai, 201102 China; 2 Shanghai Key Laboratory of Birth Defects, Shanghai, 201102 China

**Keywords:** DNA methylation, *TBX20* gene, Sp1 transcription factor, Tetralogy of Fallot

## Abstract

**Background:**

*TBX20* plays an important role in heart development; however, its epigenetic regulation in the pathogenesis of tetralogy of Fallot (TOF) remains unclear.

**Methods:**

The methylation levels of the *TBX20* promoter region in the right ventricular myocardial tissues of TOF and control samples were measured by the Sequenom MassARRAY platform. Bisulphite-sequencing PCR (BSP) was used to confirm the *TBX20* methylation of CpG sites in cells. Dual-luciferase reporter assays were performed to detect the influence of *TBX20* methylation and Sp1 transcription factors on gene activity. An electrophoretic mobility shift assay (EMSA) was used to explore the binding of the Sp1 transcription factor to the *TBX20* promoter.

**Results:**

TOF cases had a significantly lower *TBX20*_M1 methylation level than controls (median methylation: 20.40% vs. 38.73%; *p* = 0.0047). The Sp1 transcription factor, which binds to Sp1 binding sites in the *TBX20*_M1 region and promotes *TBX20* gene activity, was blocked by the methylation of Sp1 binding sites in normal controls. With decreasing methylation in the TOF cases, the Sp1 transcription factor can bind to its binding site within the *TBX20* promoter M1 region and promote *TBX20* gene expression.

**Conclusions:**

Hypomethylation of the *TBX20* promoter region was observed in the TOF cases, and the high expression of the *TBX20* gene may be caused by activated Sp1 transcription factor binding because of the decreasing methylation at the Sp1 transcription factor binding sites within *TBX20_M1.*

## Background

Tetralogy of Fallot (TOF) is characterized by pulmonary outflow tract obstruction, ventricular septal defects, overriding aortic roots, and right ventricular hypertrophy [1]. This condition occurs in one out of every 2500 live births, accounting for 11.4% of all severe congenital heart disease (CHD) cases with serious illness in the neonatal period or early infancy [2]. For the majority of cyanotic CHDs, the exact pathogenesis of TOF remains unclear. Many studies have demonstrated that the abnormal expression of cardiac-related genes influences cardiac normal development and contributes to the pathogenesis of TOF [3–5]. However, genetic changes, including chromosomal aneuploidy and single gene mutations, are only found in a small number of patients, and many TOF cases showed no sequence changes [6]. Therefore, epigenetic regulation abnormalities may be another genetic factor associated with the development of TOF.

DNA methylation is one kind of epigenetic regulation and has been studied widely. Many studies have suggested that DNA methylation is critical for early foetal development and that DNA methylation changes may be involved in the development of cardiovascular diseases [7, 8]. Short DNA sequences with a relatively high frequency of CpG sites are commonly found in the promoters of mammalian genes, and the degree of methylation at these sites is closely related to the transcription and expression status of the corresponding genes [9]. *NKX2–5* and *HAND1* are essential for heart development, and Sheng et al. demonstrated that the aberrant methylation status of the *NKX2–5* gene body and CpG island in the *HAND1* promoter regions is associated with the regulation of gene transcription in TOF patients and may contribute to the pathogenesis of TOF [10]. The *ZFPM2* gene plays an important role in heart morphogenesis and the development of coronary vessels from the epicardium. The methylation levels of the CpG island shore in the ZFPM2 promoter were significantly higher in patients with TOF than in controls and were negatively associated with significant changes in its mRNA level [11].

*TBX20* is an important transcription factor that shares a highly conserved DNA-binding region (called the T-box) and plays a crucial role in the development of CHD in humans. The *TBX20* gene has been confirmed to be expressed in the atrioventricular channel, the outflow tract and the developing right ventricle and valves during development [12, 13]. Many studies have found that nonsense mutations and missense mutations in the *TBX20* gene were associated with CHD in humans [14, 15] or led to a series of developmental abnormalities, such as compartmentalization and growth of the heart [16]. Mice with *Tbx20* homozygous loss-of-function mutations died at approximately E9.5–10.5 due to severe defects in cardiac development [17]. A partial knockdown of *TBX20* resulted in the inability of the embryonic cardiac outflow tract to effectively divide into the aorta and pulmonary arteries, resulting in right ventricular dysplasia [18]. *TBX20* is recognized as a key component of a genetic network that regulates heart development, proliferation, and differentiation during mouse cardiogenesis [19]. Cardiac *TBX20* expression was significantly increased in patients with TOF [20, 21]. Therefore, *TBX20* is essential for the development of the atrioventricular septum, outflow tract, and valve, indicating that abnormal expression of TBX20 may have a potentially important role in the development of TOF.

Specificity protein 1 (Sp1) is a member of a family of transcription factors that bind GC/GT-rich promoter elements through three C_2_H_2_-type zinc fingers that are present at their C-terminal domains [22, 23]. This molecule always binds to the promoter region of its target genes, such as *TBX20*, and can increase or decrease the transcriptional activity in response to physiological and pathological stimuli [24]. Mutation of the Sp1 binding sequence in the targeted gene may affect *TBX20* gene expression.

Although mutations in TBX20 have been found in patients with TOF, little is known about DNA methylation changes and the corresponding regulatory mechanism in TOF patients.

In this study, our aim was to explore the DNA methylation changes of *TBX20* and its regulatory mechanism in TOF patients. The results may provide important clues in understanding the aetiology of TOF.

## Methods

### Subjects and samples

Subjects with TOF disease were enrolled from the Children’s Hospital of Fudan University, Shanghai, China. These patients were diagnosed by echocardiogram and assessed for 22q11.2 deletions by a chromosomal microarray analysis (CMA) to exclude the patients with 22q11 deletion syndrome.

A total of 42 TOF patients were studied, 26 (61.9%) males and 16 (38.1%) females included, ranging in age from 1 month to 4 years (mean ± SD: 1.28 ± 1.05 years). The control cohorts consisted of autopsy specimens from normal individuals who had died from accidents, and the specimens were obtained from the Forensic Medicine Department of Fudan University, Shanghai, China. The postmortem interval (PMI) for the control samples was within 24 h. Six normal controls were recruited, including 4 (66.7%) males and 2 (33.3%) females aged 6 mo–4.5 years (mean ± SD: 1.73 ± 1.44 years). Immediately after surgical resection or autopsy, all cardiac tissue samples were taken from the right ventricular myocardial tissues and stored at − 80 °C in RNAlater® (AMBION, Inc., Austin, TX, U.S.). The characteristics of the study subjects are summarized in Table 1. The local ethics committee of Fudan University approved this study, and written informed consent was obtained from the parents or relatives of all study individuals.

**Table 1:** Demographic Characteristics of TOF Cases and Normal Controls

Characteristic	TOF(*n* = 42)	Control(*n* = 6)
Age(mean ± SD)	1.28 ± 1.05 (years)	1.73 ± 1.44 (years)
< 1	22(52.4%)	1(16.7%)
1~2	14(33.3%)	4(66.6%)
> 2	6(14.3%)	1(16.7%)
Gender		
Male(%)	26(61.9%)	4(66.7%)
Female(%)	16(38.1%)	2(33.3%)

### DNA extraction and sodium bisulphite conversion

Genomic DNA was extracted from TOF heart tissue samples and normal subjects using the QIA amp DNA Mini Kit (Qiagen, Hilden, Germany) according to the manufacturer’s instructions. A NanoDrop™ 1000 Spectrophotometer (Thermo Scientific, Wilmington, USA) was used to measure the concentration and purity of genomic DNA by absorbance at 260 and 280 nm. Genomic DNA from myocardial tissue was bisulphite-converted using an EZ Bisulfite conversion kit (Zymo Research, Orange, CA, U.S.) according to the manufacturer’s instructions. The bisulphite-converted genomic DNA was resuspended in elution buffer with 10 μl and stored at − 80 °C until analysis.

### MassARRAY analysis of TBX20 promoter methylation status

Based on base-specific cleavage and MALDI-TOF mass spectrometry, the EpiTyper MassArray (Sequenom, San Diego, CA, USA) was used for quantitative methylation analysis about the *TBX20* gene, according to the manufacturer’s recommendation. The amplicons and PCR primers required for this study were designed by the website http://epidesigner.com (Table 2). An additional T7 promoter tag was added to each reverse primer for in vivo transcription, and for each forward primer, a 10-mer tag for adjusting the melting temperature differences was added. All experiments were performed as previously described [25].

**Table 2:** Primer sequences, position, product length and CpG units used for MassArray quantitative methylation analysis

Gene	Forward primer (5′ → 3′)^a^	Reverse primer (5′ → 3′)^b^	Distance	Product	No of CpG’s	Coverage
			(bp)^c^	Length (bp)		(CpG units)
TBX20_M1	TTTGAGTGTGTATGTTAGTTTGAGTTT	CTCCTATTTTCCCTAAAAAAACCCT	−945~ − 635	311	23	22
TBX20_M2	GTGAGATTAGGTGGGGATGTTTAT	TCCTTCCTCCCTCTAAAACTAAAAA	− 214~167	382	42	32

^a^10-mer tag: cagtaatacgactcactatagggagaagg

^b^T7 promoter tag: aggaagagag was added

^c^Relative to the transcription start site

### Construction of luciferase reporter plasmids, in vitro methylation and bisulphite–PCR methylation analysis

The pGL3-TBX20_M1 plasmid was constructed by inserting the promoter region (− 945 to − 635 relative to the TSS) of *TBX20* into the pGL3-Promoter vector (Promega, Madison, WI), and the the promoter region of *TBX20* was amplified from human genomic DNA extracted from the healthy cardiac tissue using high-fidelity LA Taq PCR (TaKaRa Biotechnology). The DNA fragment containing the *TBX20* gene core promoter was amplified by PCR using the following primers:

- TBX20-KpnI-F, 5′-GGTCGGTACCCTTGAGTGTGTATGTCAGCCTGAGT-3′;
- TBX20-XhoI-R, 5′-GGTCCTCGAGCTCCTATTTTCCCTAGAGGGACCCT-3′.

The fidelity of the DNA sequence inserted into pGL3-Promoter was verified by direct sequencing. Similarly, the Mut-pGL3-TBX20_M1 plasmid was constructed, and the mutated sequence in the plasmid was as follows:

- Mut-TBX20-F, 5′-GTAGAATCAAAAATTTCACACTTCG-3′;
- Mut-TBX20-R, 5′-CGAAGTGTGAAATTTTTGATTCTAC-3′.

The Me-pGL3-TBX20_M1 plasmid was methylated by incubating pGL3-TBX20_M1 with M.SssI (New England BioLabs, Beijing, China) in the presence of 160 μmol/l S-adenosylmethionine for 3 h at 37 °C. BSP was used to confirm the methylation status. pGL3-TBX20_M1 was subjected to sodium bisulphite treatment using the EZ DNA methylation kit (Zymo Research, Orange, CA, U.S.). The bisulphite-converted DNA fragment containing the *TBX20* gene core promoter was amplified by PCR using the following primers:

- TBX20-BSP-F, 5′-TTTAGGTTGAGTAGGGTTAATAGGA-3′;
- TBX20-BSP-R, 5′-CCTAAAAAAACCCTAAAAAATAAAAC-3′.

Following PCR amplification, 5 μl of each PCR product was verified on a standard 2.0% agarose gel, and the rest of the PCR product was purified with the AxyPrep DNA Gel Extraction Kit (Axygen, Union City, CA) according to the manufacturer’s instructions. Purified PCR products were cloned into a pGEM-TEasy vector (Promega, Madison, WI) and then transformed into DH5α competent cells (TIANGEN, Beijing, China). After incubation for 12 h at 37 °C, blue/white and ampicillin screening was performed. Plasmids were extracted from 10 white colonies of each sample screened and purified, and then sequenced using T7 primers.

BIQ Analyzer software (Sarbrücken, Germany) was used to analyse the sequencing data obtained from BSP and cloning-based sequencing. The percent methylation of each CpG site in a screened sample was calculated as the number of methylated CpG sites in the total number of observed sequenced clones. The average of the methylation status of each CpG site in the DNA region was the overall methylation status of a specific region in a screened sample.

### Cell culture, transient transfection and dual-luciferase reporter assays

HEK293T cells (human embryonic kidney) and HL-1 cells (mouse cardiac muscle) were grown in Dulbecco’s modified Eagle’s medium (Gibco™) supplemented with 10% foetal bovine serum (Gibco™) and 1 × Pen/Strep (Thermo Fisher Scientific). All cells were cultured at 37 °C and 5% CO_2_. All transient transfections were performed with Lipofectamine 3000 (Invitrogen™) according to the manufacturer’s protocol. Both cells were plated in 96-well plates and transfected with 100 ng of pGL3-Basic, pGL3-Promoter, pGL3-TBX20_M1 (unmethylated) and Me-pGL3-TBX20_M1 (methylated). For cotransfection luciferase assays, 100 ng of pGL3-Basic, pGL3-TBX20_M1 (unmethylated), Mut-pGL3-TBX20_M1 (mutated) and Me-pGL3-TBX20_M1 (methylated) and 100 ng transcription factor expression vectors were cotransfected into cells. The empty vector pcDNA3.1(+) was added to equalize the final DNA content among each well. The pGL3-Basic vector containing no promoter sequences was used as the negative control. To normalize the luciferase activity, the pRL-TK plasmid (Promega) containing the Renilla luciferase gene was cotransfected with these plasmids. The luciferase activity was measured by the Dual-Luciferase Reporter Assay System (Promega) until cells were harvested 48 h after transfection. Triplicate samples were performed and the assays were repeated in at least three independent experiments.

### EMSA (electrophoretic mobility-shift assay) and shift-western blotting

HEK293T cells were transfected with the pcDNA3.1-Sp1 expression plasmid. The nuclear extracts were obtained using nuclear and cytoplasmic extraction reagents (Thermo Scientific™) according to the manufacturer’s protocol. The concentration of the protein was measured by a BCA protein assay kit (Pierce™). Sp1 antibodies were obtained from Abcam. Sp1 overexpression was verified by Western blotting. Sequence-specific probes (Generay, Shanghai, China) were synthesized and annealed into double strands. A biotin-labelled oligonucleotide probe containing a Sp1 binding site (Biotin-TBX20–708/− 684) and a mutant biotin-labelled oligonucleotide probe were used to verify Sp1 binding specificity (Biotin-TBX20–708/− 684mut), and an unlabelled oligonucleotide probe (TBX20–708/− 684), a mutant unlabelled oligonucleotide probe (TBX20–708/− 684mut), and a methylated oligonucleotide (Me-TBX20–708/−684) were also used. The sequences of these oligonucleotide probes are summarized in Table 3.

**Table 3:** The sequences of double-stranded oligonucleotide probes used for EMSA

Probe	Sequences(5′-3′)
Biotin-TBX20–708/−684-F	GTAGAGCTCCGCGCCCTGACCTTCG
Biotin-TBX20–708/−684-R	CGAAGGTCAGGGCGCGGAGCTCTAC
Biotin-TBX20–708/−684mut-F	GTAGAATCAAAAATTTCACACTTCG
Biotin-TBX20–708/−684mut-R	CGAAGTGTGAAATTTTTGATTCTAC
Unlabeled-TBX20–708/−684mut-F	GTAGAATCAAAAATTTCACACTTCG
Unlabeled-TBX20–708/−684mut-R	CGAAGTGTGAAATTTTTGATTCTAC
Me-TBX20–708/−684-F	GTAGAGCTCCGCGCCCTGACCTTCG
Me-TBX20–708/−684-R	CGAAGGTCAGGGCGCGGAGCTCTAC

The binding ability of Sp1 to *TBX20* was detected by EMSA with a Scientific Light-Shift EMSA KIT (Thermo Scientific™) according to the manufacturer’s protocols. Briefly, 20 fmol of biotin-labelled oligonucleotide probes was added to the reaction, and the control group was supplemented with a 100-fold excess of competitor/competitor-mut oligonucleotide probes. After incubation, the mixtures were conducted on polyacrylamide gels and transferred onto nylon membranes and photographed with a Fuji film Las3000 Luminescent Image Analyzer (Fuji Life Sciences, Tokyo, Japan).

Shift-Western blotting was performed by transfer of DNA-protein complexes from a polyacrylamide gel to a nitrocellulose membrane using a Trans-Blot SD semi-dry cell (Bio-Rad). The DNA-protein complexes on the membrane were visualized by staining with a solution of 1% Ponceau (Sigma) and 1% acetic acid. Membranes were blocked with blocking buffer (1× Tris-buffered saline (TBS) containing 5% nonfat milk powder and 0.1% Tween) for 1 h at room temperature. Membranes were incubated with blocking buffer containing primary Sp1 antibody at 4 °C overnight. The blots were washed and incubated with secondary antibodies conjugated to horseradish peroxidase. Immunostaining was visualized using a chemiluminescent detection kit (Thermo Fisher Scientific™) as described by the manufacturer.

## Results

### Methylation status for the *TBX 20* gene in TOF patients and controls

**Fig. 1:**
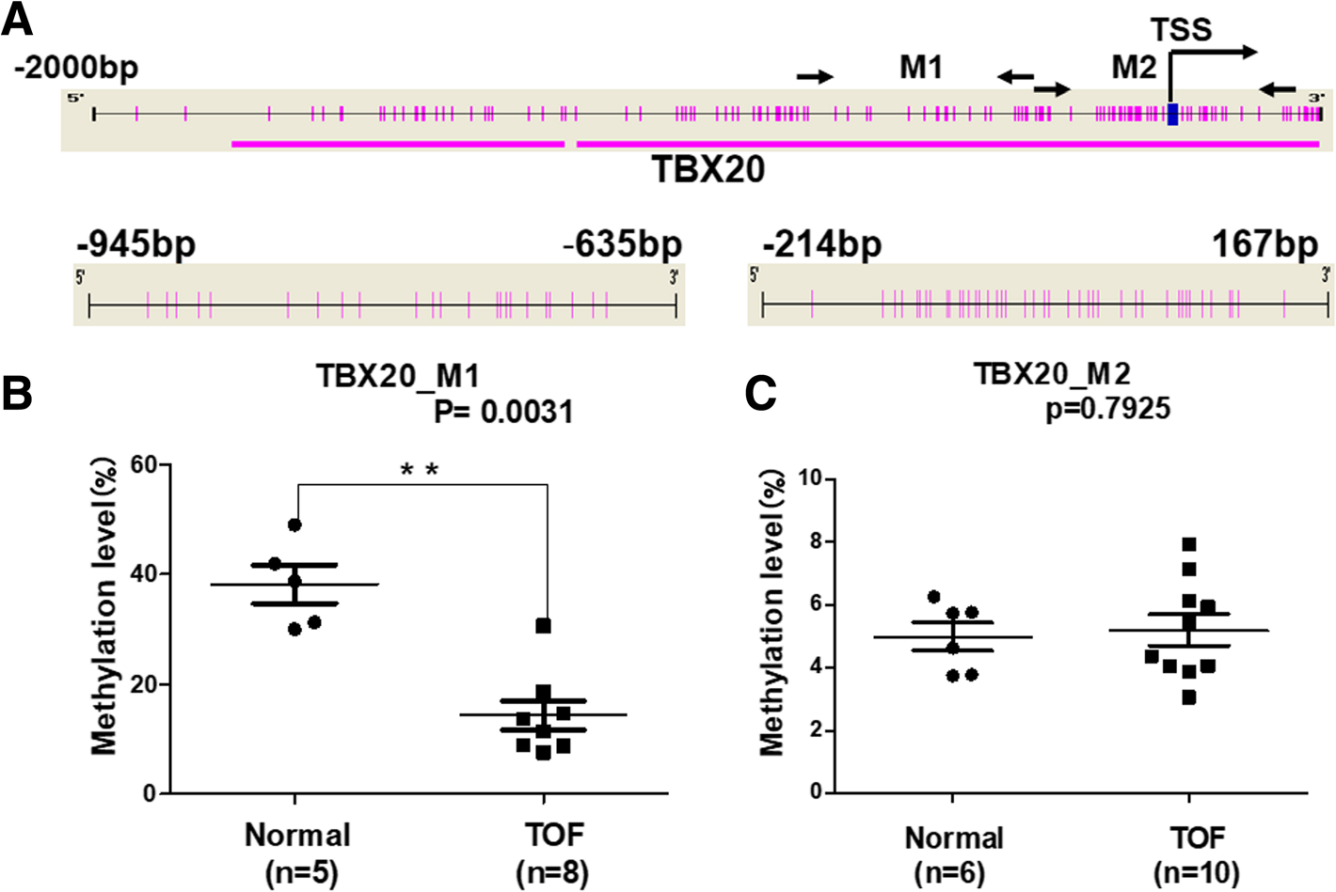
The schematic of the *TBX20* promoter and its methylation status. **a** Schematic representation of the distribution of *TBX20_M1* and *TBX20_M2* in the promoter region; **b** The methylation levels for *TBX20_M1* between 5 normal and 8 TOF subjects; **c** The methylation levels for *TBX20_M2* between 6 normal and 10 TOF subjects*.* All CpG sites of the *TBX20* promoter are represented as pink ticks, *P < 0.05, **P < 0.01, ***P < 0.001 (Mann-Whitney test)

The methylation status of the *TBX20* gene promoter was determined by the Sequenom MassARRAY platform. Two amplicons in the *TBX20* promoter region (*TBX20_*M1: − 945 bp ~ − 635 bp, *TBX20_* M2: − 159 bp ~ 217 bp, Fig. 1a) were analysed in 10 patients with TOF and 6 matched controls. For the methylation data, we performed a strict quality control as follows: The unreliable CpG units (failing to produce data from more than 30% of samples) and unreliable samples (missing more than 30% of the data points) were discarded.

As shown in Fig. 1b, the methylation level of *TBX20*_M1 was significantly lower, with a median of 12.6% (IQR: 8.8–17.8%, *N* = 8; a 6-month-old male and a 5-month-old female had no effective methylation data) in TOF patients, compared to a median of 38.7% in controls (IQR: 30.6–45.5%; *p* = 0.0031, *N* = 5; one 6-month-old male had unreliable samples). Methylation levels in TBX20_M2 were not significantly different between the 10 TOF and 6 control subjects (4.9% vs. 5.2%; *p* = 0.7925, Fig. 1c).

**Fig. 2:**
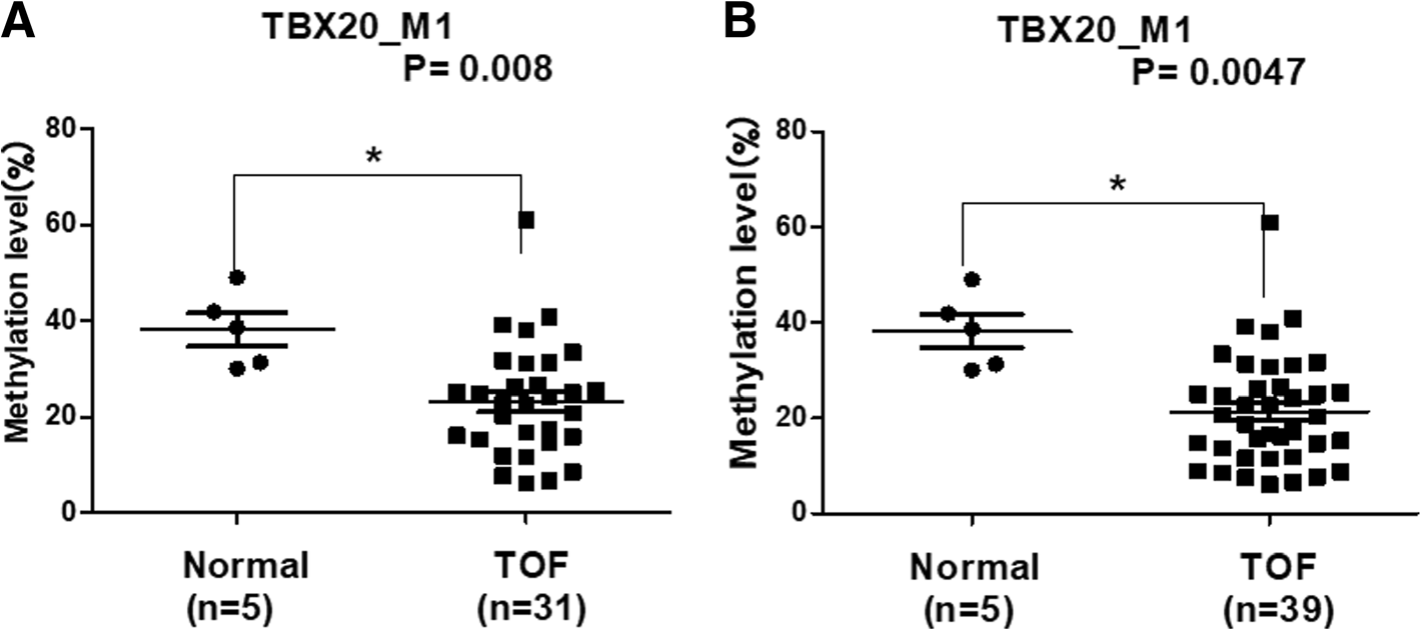
The methylation levels for the *TBX20_M1* region. **a** The methylation levels for *TBX20_M1* between 5 normal and 31 TOF subjects; **b** The methylation levels for *TBX20_M1* between 5 normal and 39 TOF subjects*.* *P < 0.05, **P < 0.01, ***P < 0.001 (Mann-Whitney test)

The different methylation levels of *TBX20*_M1 were then validated in 32 patients with TOF. As shown in Fig. 2a, the methylation level of *TBX20*_M1 was significantly lower in 31 patients with TOF (one 36-month-old male had no effective methylation data), with a median value of 22.7% (IQR: 15.3–31.0%) compared to 38.7% (IQR: 30.6–45.5%) in 5 controls (*p* = 0.008). Furthermore, combined with the methylation data in the initial 8 TOF cases, a significantly lower *TBX20*_M1 methylation level was observed in 39 TOF cases, with a median value of 20.4% (IQR: 11.9–26.5%) compared to 38.7% (IQR: 30.6–45.5%) in 5 controls (*p* = 0.0047, Fig. 2b).

### The impact of *TBX20_ M1* region methylation on gene transcription activity

**Fig. 3:**
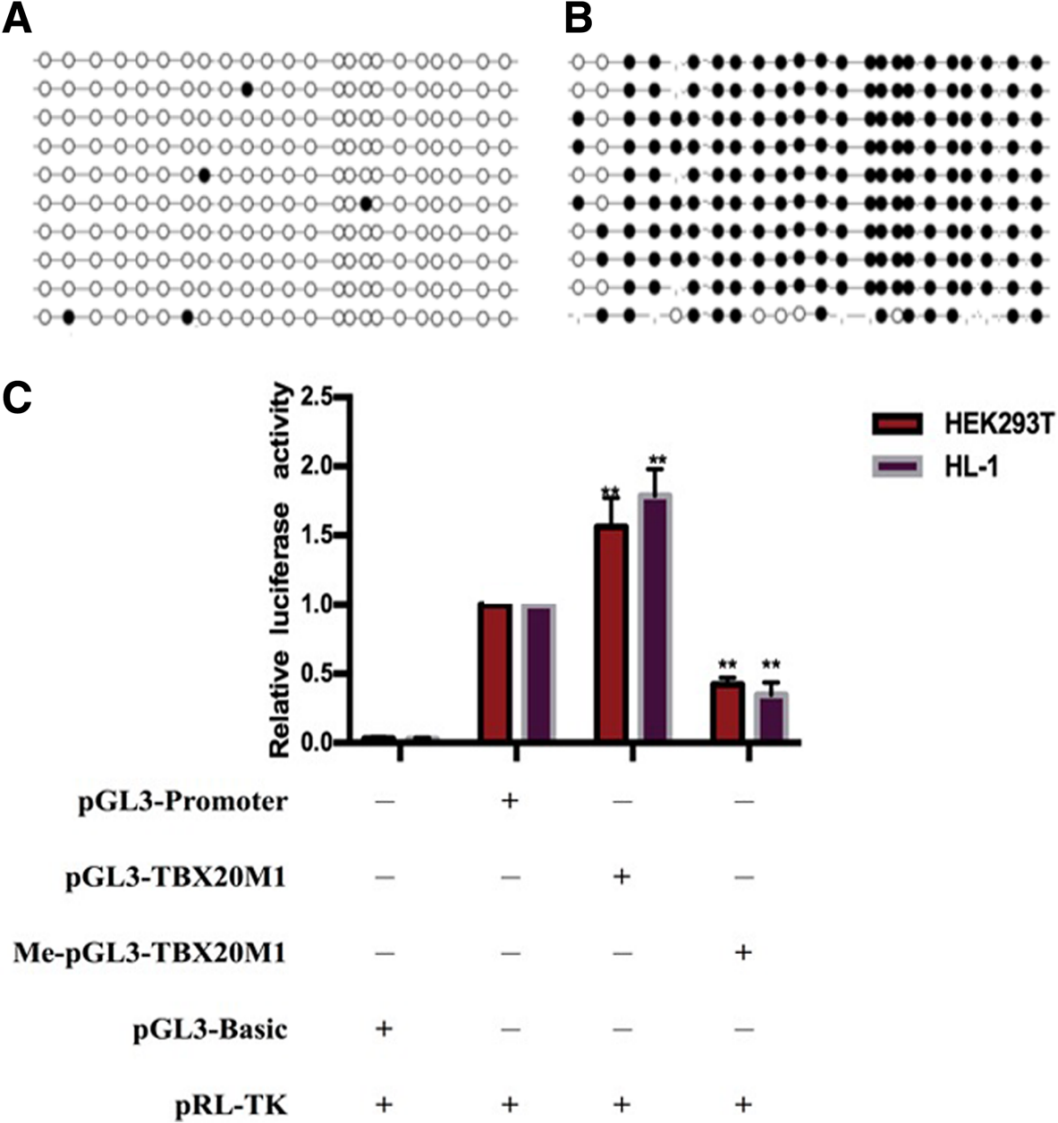
The impact of *TBX20_ M1* region methylation on gene transcription activity. **a** The methylation status of BSP for the pGL3-TBX20_M1 region; **b** The methylation status of BSP for the Me-pGL3-TBX20_M1 region; **c** The luciferase activity assays for pGL3-Basic, pGL3-Promoter, pGL3-TBX20_M1 and Me-pGL3-TBX20_M1 in HEK 293 T and HL-1 cell lines, and the data are shown as the fold increase or decrease over the luciferase activity of pGL3-Promoter. The white circle represents unmethylated CpG sites, the black circle represents methylated CpG sites, *P < 0.05, **P < 0.01 (Mann-Whitney test)

To investigate whether *TBX20*_M1 region methylation influences gene transcription activity, we performed a luciferase assay using HEK293T and HL-1 cells. pGL3-TBX20_M1 was generated by inserting the TBX20 promoter M1 region (− 945 bp ~ − 635 bp) into pGL3-Promoter. The methylated pGL3-TBX20_M1 was produced by treating pGL3-TBX20_M1 with CpG methyl-transferase (M.SssI) in vitro (Fig. 3a, b). The pGL3-Basic, pGL3-Promoter, pGL3-TBX20_M1 and Me-pGL3-TBX20_M1 vectors were subsequently transfected into HEK293T and HL-1 cell lines with an SV40 promoter-driven firefly luciferase reporter. As shown in Fig. 3c, the luciferase activity of pGL3-TBX20_M1 was significantly higher than that of pGL3-Promoter, indicating that the *TBX20* promoter M1 region (− 945 bp ~ − 635 bp) can promote gene transcription activity. Compared to pGL3-TBX20_M1, Me-pGL3-TBX20_M1 significantly reduced luciferase activity by nearly fourfold. These findings suggested that the *TBX20_M1* region can increase gene transcription activity and that methylation changes in this region are responsible for gene transcription activity.

### The Sp1 transcription factor promotes gene transcription activity by binding to the *TBX20_M1* region

**Fig. 4:**
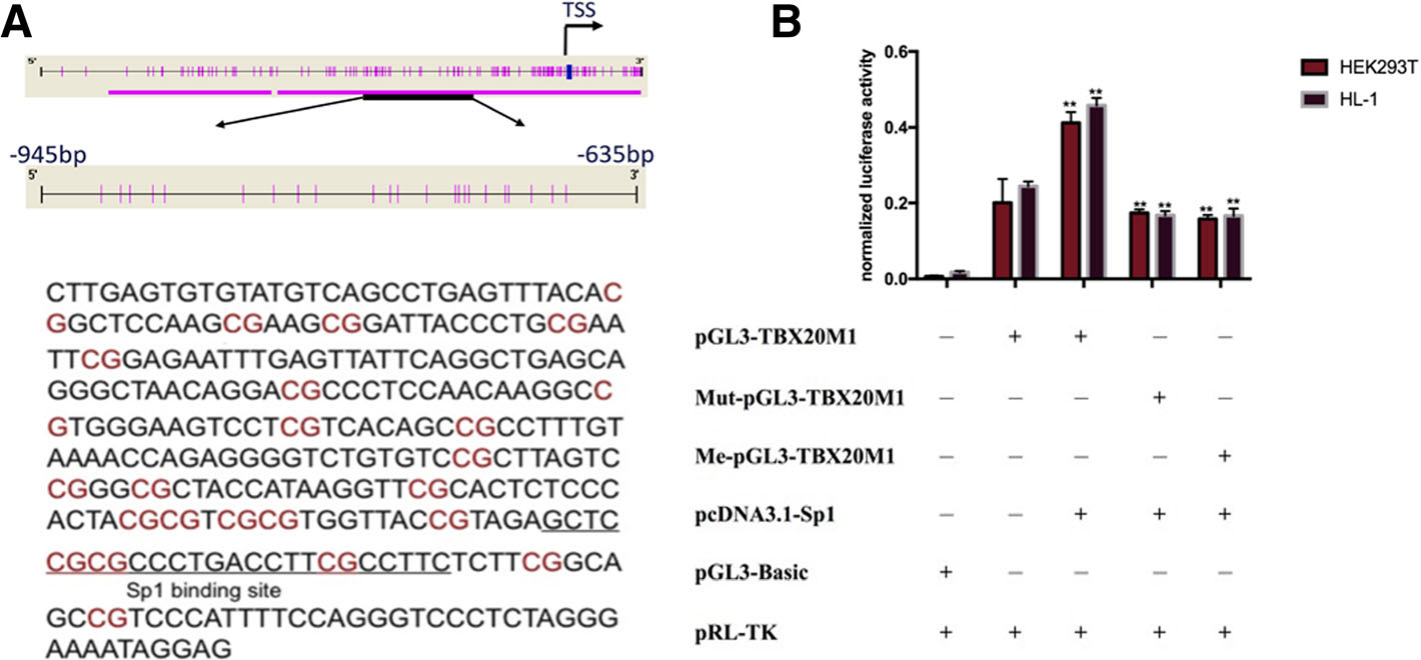
Sp1 promotes gene transcription activity by binding to the *TBX20_M1* region. **a** Schematic of the DNA sequence of TBX20_M1 and Sp1 binding sites in this region. **b** Luciferase activity assays for pGL3-Basic, pGL3-TBX20_M1, pcDNA3.1-Sp1, Me-pGL3-TBX20_M1 and Mut-pGL3-TBX20_M1 in HEK 293 T and HL-1 cell lines. *P < 0.05, **P < 0.01 (Mann-Whitney test)

To explore the mechanism underlying the effect of the *TBX20_*M1 region on gene transcription activity, we analysed the *TBX20* promoter M1 region sequence by a TF search (http://www.cbrc.jp/research/db/ TFSEARCH.html) and the JASPAR database(http://jaspar.binf.ku.dk/cgi-bin/jaspar_db.pl?-rm=browse&db=core&tax_group=vertebrates) and found potential transcription factor binding sites for Sp1 (Fig. 4a). We constructed the Sp1 expression plasmid pcDNA3.1-Sp1 and cotransfected HEK293T and HL-1 cells with a luciferase reporter gene driven by PGL3-Basic, pGL3-TBX20_M1, Mut-pGL3-TBX20_M1 and Me-pGL3-TBX20_M1. As shown in Fig. 4b, the luciferase activity driven by pGL3-TBX20_M1 was significantly higher than that of pGL3-Basic. When the Sp1 transcription factor was present, pGL3-TBX20_M1 significantly increased luciferase activity. However, Mut-pGL3-TBX20_M1, which carried the mutated Sp1 transcription factor binding sites, showed significantly reduced luciferase activity. These findings indicated that the Sp1 transcription factor can bind to the corresponding binding sites in the *TBX20_M1* region and promote gene transcription activity. When pcDNA3.1-Sp1 and Me-pGL3-TBX20_M1 were cotransfected into HEK293T and HL-1 cell lines with an SV40 promoter-driven firefly luciferase reporter, the gene activity was significantly reduced, consistent with the results of Mut-pGL3-TBX20_M1, suggesting that the methylation of Sp1 transcription factor binding sites in the *TBX20_M1* region inhibited gene activity.

### Effect of *TBX20_M1* methylation on Sp1 transcription factor binding

**Fig. 5:**
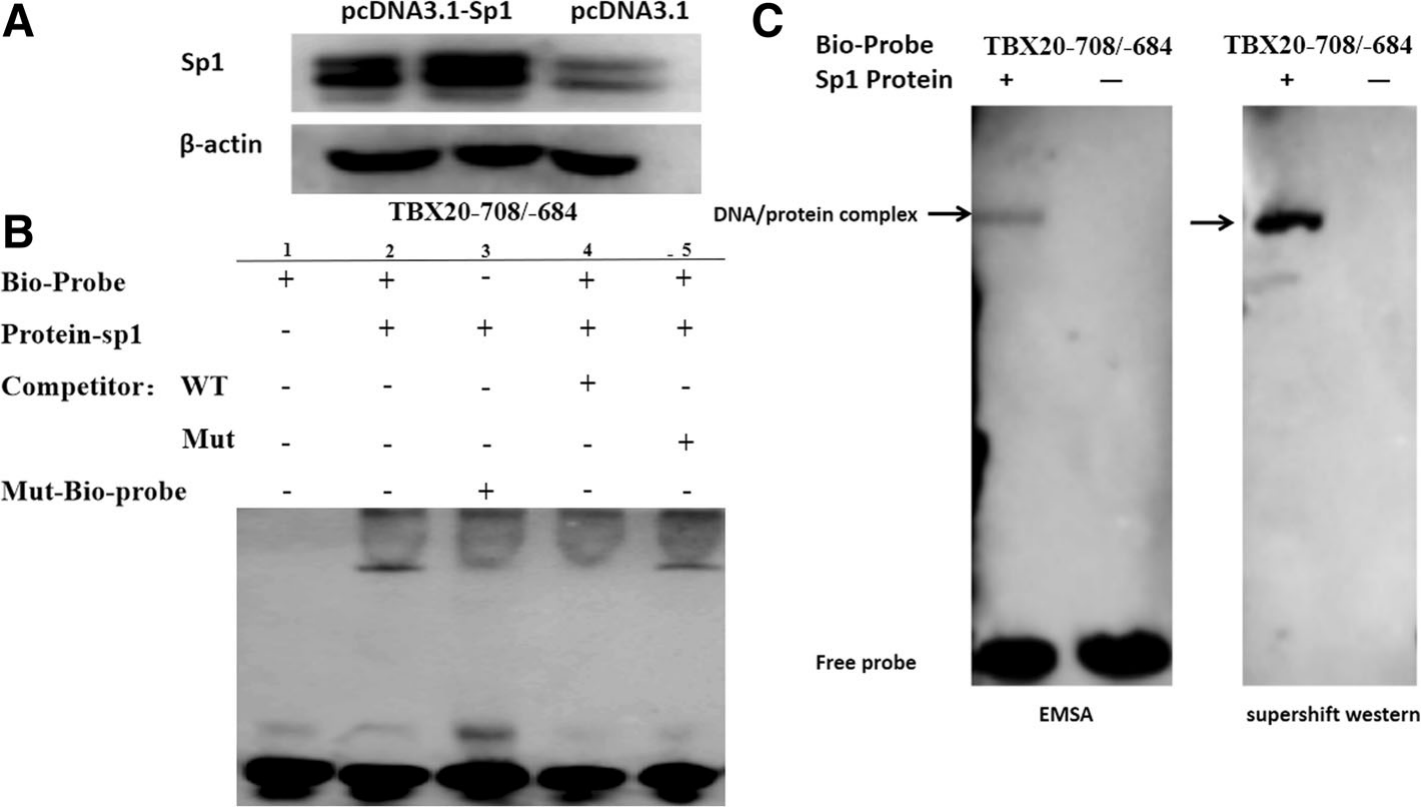
Sp1 transcription factor binding to the *TBX20_M1* region. **a** Sp1 transcription factor overexpression in HEK 293 T cell lines; **b** EMSAs confirmed the binding of the Sp1 protein to the *TBX20_M1* region. Lane 1 contains only the Bio-labelled probe (*TBX20*_-708 bp/− 684 bp) alone; Lane 2: Bio-labelled probe + Sp1 protein in HEK 293 T cells; Lane 3: Mut-Bio-labelled probe + Sp1 protein in HEK 293 T cells; Lane 4: Bio-labelled probe + unlabelled competitor WT probe +Sp1 protein in HEK 293 T cells; Lane 5: Bio-labelled probe + unlabelled competitor Mut probe +Sp1 protein in HEK 293 T cells; **c** EMSA and super-shift western blot analyses confirmed the Sp1 protein binding. The arrows indicate the complex of Bio-labelled *TBX20* probe and Sp1 protein

To determine whether the Sp1 transcription factor binds to the *TBX20_M1* region, we overexpressed Sp1 in HEK293T cell lines (Fig. 5a) and performed EMSAs using Sp1 protein from transfected whole cell lysates and a biotin-labelled probe (TBX20_-708 bp/− 684 bp) or mutated biotin-labelled probe.

As shown in Fig. 5b, the biotin-labelled probe can bind to the Sp1 transcription factor in vitro (Fig. 5b, lane 2). However, this reaction was not observed when the mutated biotin-labelled or wild-type competition probe was added, indicating that the binding was blocked (Fig. 5b, lane 3, lane 4). When the mutant competition probe was added, the binding of the Sp1 transcription factor to the biotin-labelled probe (TBX20_-708 bp/− 684 bp) was not influenced (Fig. 5b, lane 5). These results indicated that Sp1 can bind to the *TBX20_M1* region. A super-shift western blot was performed to verify that the banding is actually caused by Sp1 protein (Fig. 5c).

**Fig. 6:**
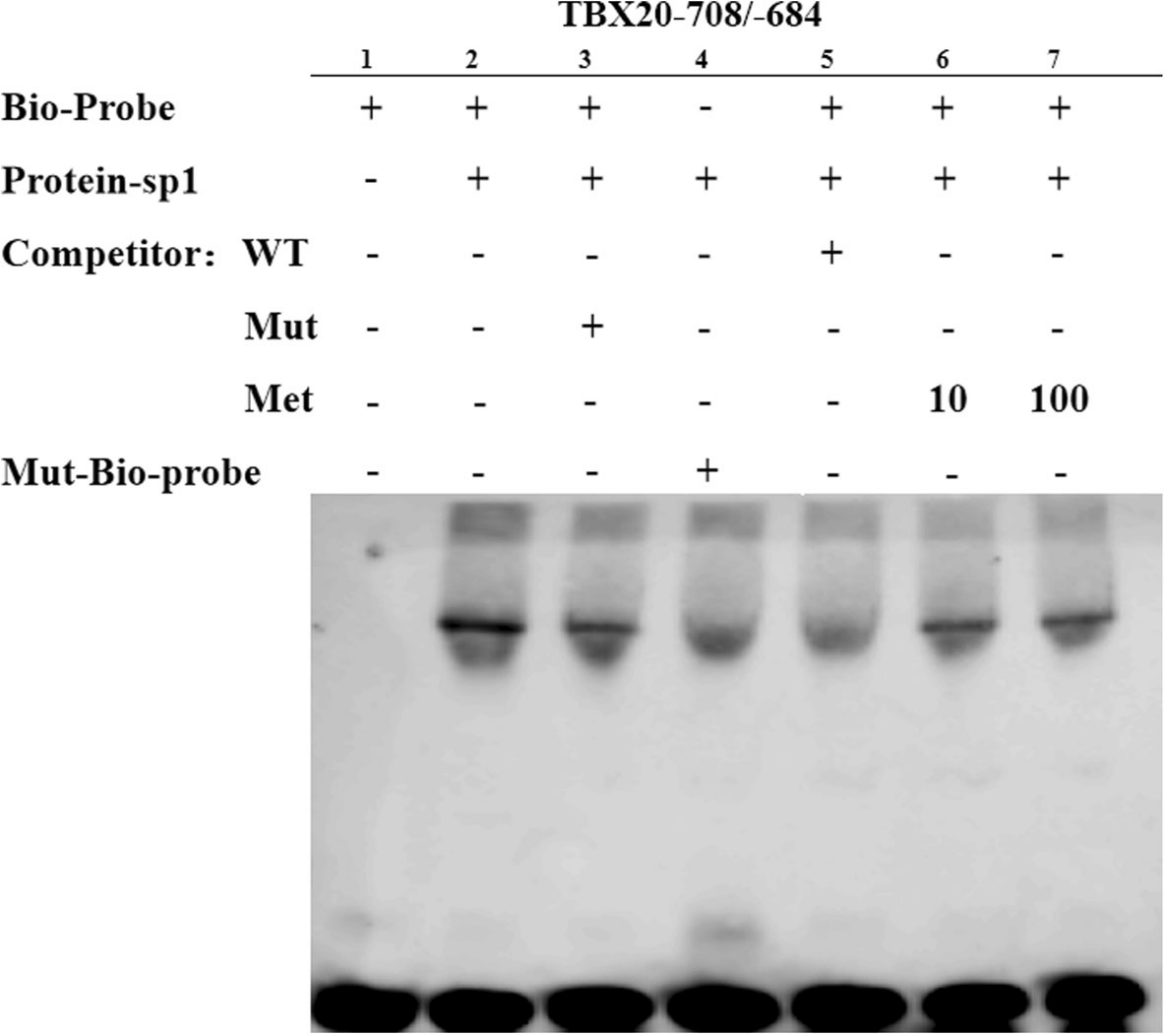
The influence of *TBX20_M1* methylation on Sp1 transcription factor binding. Lane 1 contains only the Bio-labelled probe (*TBX20*_-708 bp/− 684 bp) alone; Lane 2: Bio-labelled probe + Sp1 protein in HEK 293 T cells; Lane 3: Bio-labelled probe + Sp1 protein + unlabelled competitor Mut probe; Lane 4: Mut-Bio-labelled probe + Sp1 protein in HEK 293 T cells; Lane 5: Bio-labelled probe + Sp1 protein + unlabelled competitor WT probe; Lane 6: Bio-labelled probe + Sp1 protein + unlabelled competitor Met probe (10-fold); Lane 7: Bio-labelled probe + Sp1 protein + unlabelled competitor Met probe (100-fold)

Furthermore, we performed EMSA to confirm whether *TBX20_M1* methylation influences Sp1 binding. As shown in Fig. 6, the binding of Sp1 to the biotin-labelled probe was observed in the presence of Bio-Probe (Fig. 6, lane 2) and blocked by the presence of the Mut-Bio-Probe or wild-type competition probe (Fig. 6, lane 4,5). When 10-fold or 100-fold excess unlabelled methylated competition probe was added, the binding reaction was not influenced (Fig. 6, lane 6,7), consistent with the results of the mutant competition probe analysis (Fig. 6, lane 3). These findings showed that methylation at the Sp1 binding sites within *TBX20-M1* blocks Sp1 binding.

## Discussion

Previous studies have demonstrated a potentially significant part of the *Tbx20* gene in mouse embryo heart development [16, 26], and abnormal cardiac TBX20 expression was identified to be associated with the pathogenesis of CHD [27]. Human *TBX20* mutations are associated with a complex spectra of cardiac development and dysfunction, including TOF [14, 28], but the mutation frequency is low. In addition to DNA sequence variations in genes, epigenetic mechanisms that control gene expression without DNA sequence changes have been found to play necessary parts in cardiac development [29–31]. Abnormal changes in methylation are always associated with aberrant gene expression and contribute to disease development. Many studies on the methylation of target genes in diseases have clarified the relationship between aberrant methylation, including hypermethylation or hypomethylation, and the pathogenesis of diseases [32]. Many studies have focused on the methylation change of CpG islands or short fragment DNA with a high content of CpG sites in the promoter, which is critical for transcriptional regulation [33]. In our previous studies, we found that hypomethylation levels of LINE-1 (long interspersed nucleotide elements-1) were associated with an increased risk of TOF, and methylation of the promoters of some genes contributed to the downregulation of genes in the myocardium of TOF patients [25, 34, 35]. In this study, we observed that the *TBX20-M1* region in the *TBX20* promoter was hypomethylated in the myocardium of TOF patients, was located in an important regulatory region of the *TBX20* promoter and played a key role in transcriptional activity. The overexpression of *TBX20* has been observed in TOF patients. We concluded that the hypomethylation level of the *TBX20-M1* region may be correlated with the overexpression of the *TBX20* gene.

Many studies have confirmed that the methylation of genomic DNA contributes to transcriptional repression, either by blocking the binding of transcriptional activators to cognate DNA sequences or by recruiting methyl-CpG-binding proteins, which recruit chromatin-remodelling machinery to induce the formation of an inhibitory chromatin structure [36, 37]. CpG site-specific methylation was found to alter the binding affinities of specific transcription factors that can activate or repress transcription [38]. Tihomira D et al. found that although methylation of all CpG sites was responsible for EphA5 promoter inactivity, low levels of methylation resulted in differential activation or repression of EphA5 promoter activity, depending on the methylation status of the sites [39].

To investigate whether the overexpression of the *TBX20* gene was caused by aberrant methylation of the M1 region CpG sites of the *TBX20* promoter, dual-luciferase reporter assays were performed in combination with in vitro methylation assays. Following in vitro methylation, the transcriptional activity of pGL3-TBX20_M1 was significantly decreased, which indicate that the methylation level of *TBX20-M1* region CpG sites had a negative effect on the regulation of its transcriptional activity. Regarding the DNA methylation regulatory mechanism of the *TBX20-M1* region CpG sites, we suggest that methylation at the *TBX20-M1* region CpG sites influences transcription factor binding and leads to TBX20 expression changes. In the current study, the in silico analysis found that the *TBX20* promoter M1 region contains potential binding sites for Sp1. Sp1 is a transcription regulator that plays an important role in various cellular functions, such as apoptosis and invasion, and can promote or inhibit the expression of its target gene by binding to GC-rich motifs [40, 41]. We observed that Sp1 can bind to the *TBX20* promoter M1 region and increase gene transcriptional activity. Interestingly, methylation of the *TBX20* promoter M1 region can block the binding of the Sp1 protein in the cell and inhibit gene activity. These findings suggested that the Sp1 transcription factor can regulate *TBX20* gene expression based on the methylation status of the *TBX20_M1* region. If the *TBX20_M1* region showed decreased methylation levels, Sp1 could bind to this region and activate gene expression, which is consistent with the results observed in the cardiac tissue of TOF patients.

To further validate the fidelity of Sp1 binding to the promoter of the *TBX20* gene, we performed EMSA and super-shift western blotting in vitro and found that Sp1 can directly bind to the *TBX20_M1* region, and super-shift western blotting proved that the protein band is actually Sp1 with an antibody. Furthermore, we performed EMSA to confirm that methylation at the Sp1 binding sites within *TBX20-M1* blocks Sp1 transcription factor binding and influences gene expression. Although our results provide epigenetic evidence that Sp1 can regulate the expression of the *TBX20* gene in a methylation-dependent manner, the specific mechanism of demethylation and regulation needs to be further investigated.

However, the limitation of this study was that we did not gain sufficient fully matched samples because it is arduous to collect heart tissue samples from healthy controls and TOF patients, which may have an effect on the veracity of the methylation analysis. Larger ample sizes are needed to confirm our findings in future studies. Moreover, although we found that aberrant methylation of TBX20 may play a critical part in the development of TOF, we were unable to determine whether the observed methylation changes occurred after the heart had formed or after the onset of heart defects. We also could not ensure whether these changes reflect the disease physiology or the cause of disease aetiology since the development of TOF long predated the methylation measurement. All of these related issues need to be further explored using cell lines or animal models in future studies.

## Conclusion

In summary, our results suggested that the *TBX20* promoter M1 region was hypomethylated in the myocardium of TOF patients and that demethylation at the *TBX20* promoter may be responsible for the overexpression in the myocardium of patients with TOF. The high expression of the *TBX20* gene in TOF patients may be regulated by an epigenetic mechanism that decreased methylation at the Sp1 transcription factor binding sites within the *TBX20_M1* region, increased activated Sp1 transcription factor binding and promoted *TBX20* gene expression. These findings will provide new epigenetic insight into the pathogenesis of TOF.

## Abbreviations

BSP: Bisulfite-Sequencing PCR
CHD: Congenital heart disease
EMSA: Electrophoretic mobility shift assay
IQR: Interquartile range
RVOT: Right ventricular outflow tract
TOF: Tetralogy of Fallot
